# The amelioration of phagocytic ability in microglial cells by curcumin through the inhibition of EMF-induced pro-inflammatory responses

**DOI:** 10.1186/1742-2094-11-49

**Published:** 2014-03-19

**Authors:** Gen-Lin He, Yong Liu, Min Li, Chun-Hai Chen, Peng Gao, Zheng-Ping Yu, Xue-Sen Yang

**Affiliations:** 1 Key Laboratory of Medical Protection for Electromagnetic Radiation Ministry of Education, Third Military Medical University, 30 Gaotanyan Street, Chongqing 400038, PR China; 2 Institute of Tropical Medicine, Third Military Medical University, Chongqing 400038, PR China; 3 Southwest Eye Hospital, Southwest Hospital, Third Military Medical University, Chongqing 400038, PR China

## Abstract

**Background:**

Insufficient clearance by microglial cells, prevalent in several neurological conditions and diseases, is intricately intertwined with MFG-E8 expression and inflammatory responses. Electromagnetic field (EMF) exposure can elicit the pro-inflammatory activation and may also trigger an alteration of the clearance function in microglial cells. Curcumin has important roles in the anti-inflammatory and phagocytic process. Here, we evaluated the ability of curcumin to ameliorate the phagocytic ability of EMF-exposed microglial cells (N9 cells) and documented relative pathways.

**Methods:**

N9 cells were pretreated with or without recombinant murine MFG-E8 (rmMFG-E8), curcumin and an antibody of toll-like receptor 4 (anti-TLR4), and subsequently treated with EMF or a sham exposure. Their phagocytic ability was evaluated using phosphatidylserine-containing fluorescent bioparticles. The pro-inflammatory activation of microglia was assessed via CD11b immunoreactivity and the production of tumor necrosis factor-α (TNF-α), interleukin-6 (IL-6), interleukin-1β (IL-1β) and nitric oxide (NO) via the enzyme-linked immunosorbent assay or the Griess test. We evaluated the ability of curcumin to ameliorate the phagocytic ability of EMF-exposed N9 cells, including checking the expression of MFG-E8, α_v_β_3_ integrin, TLR4, nuclear factor-κB (NF-κB) and signal transducer and activator of transcription 3 (STAT3) using Western blotting.

**Results:**

EMF exposure dramatically enhanced the expression of CD11b and depressed the phagocytic ability of N9 cells. rmMFG-E8 could clearly ameliorate the phagocytic ability of N9 cells after EMF exposure. We also found that EMF exposure significantly increased the secretion of pro-inflammatory cytokines (TNF-α, IL-6 and IL-1β) and the production of NO; however, these increases were efficiently chilled by the addition of curcumin to the culture medium. This reduction led to the amelioration of the phagocytic ability of EMF-exposed N9 cells. Western blot analysis revealed that curcumin and naloxone restored the expression of MFG-E8 but had no effect on TLR4 and cytosolic STAT3. Moreover, curcumin significantly reduced the expression of NF-κB p65 in nuclei and phospho-STAT3 (p-STAT3) in cytosols and nuclei.

**Conclusions:**

This study indicates that curcumin ameliorates the depressed MFG-E8 expression and the attenuated phagocytic ability of EMF-exposed N9 cells, which is attributable to the inhibition of the pro-inflammatory response through the NF-κB and STAT3 pathways.

## Background

The application of electromagnetic fields (EMFs) is ubiquitous in modern society [1]. Several epidemiological and experimental studies have shown that EMF exposure may have detrimental effects on cognitive function [2,3] and may increase the risk of neurological diseases, such as gliomas [4,5] and Alzheimer’s disease [6,7]. EMF exposure has also been demonstrated to induce strong glial reactivity in different brain regions [8-10]. We previously observed that EMF exposure initiated the pro-inflammatory activation of microglial cells [11]. However, to our knowledge, there is no evidence for how EMF exposure induces depression of microglial phagocytosis.

The efficient removal of pathogens and cell debris by activated microglial cells is fundamental for central nervous system (CNS) development and homeostasis [12,13]. As an essential factor in the phagocytic process, milk-fat globule EGF factor-8 (MFG-E8), which has a phosphatidylserine (PS) binding domain, has been confirmed as a binding bridge between apoptotic cells and integrin α_v_β_3_ on the surface of phagocytes [14,15]. A strong pro-inflammatory response was found in MFG-E8^−/−^ mice [16]. Lipopolysaccharide (LPS), a potent stimulator of aberrant innate immune functions during inflammation, significantly decreases endogenous MFG-E8 levels *in vivo* and *in vitro* via the toll-like receptor 4 (TLR4) signaling pathway [17]. In contrast, the MFG-E8-mediated phagocytosis of apoptotic cells results in an inhibition of inflammation via the MAPK and nuclear factor-κB (NF-κB) signaling pathways following the endotoxin response [18]. Thus, these findings indicate that the role of MFG-E8 in regulating the immune reaction of microglial phagocytosis and pro-inflammatory responses could depend on the types of stimulus.

During the clearance process, it has been confirmed that curcumin has an important role as an immunomodulator in inhibiting neurotoxicity and increasing the phagocytosis index [19,20]. Previous studies showed that curcumin attenuates microglial migration and triggers a phenotype with anti-inflammatory and neuroprotective properties [21]. The activation of microglial cells can be effectively inhibited by incubating them with curcumin, which decreases the production of NO [22] and pro-inflammatory cytokines, such as IL-1β, IL-6 and TNF-α [23]. Moreover, curcumin is able to suppress the inflammatory signaling of NF-κB and the signal transducers and activators of transcription (STATs) in LPS-stimulated microglial cells [24,25]. However, the exact molecular mechanisms underlying the salutary effect of curcumin on microglial phagocytosis in inflammation remain largely unknown.

N9 cells were used because they have been proven to mimic primary microglia [26,27] and exhibit robust pro-inflammatory and phagocytic responses [28]. In this study, we tested the following questions. Does EMF exposure depress microglial phagocytosis during inflammation? If EMF depresses phagocytosis, does curcumin directly regulate the depression of microglial phagocytosis or through its anti-inflammatory effects? We demonstrated that the inhibition of microglial pro-inflammatory responses by curcumin restores MFG-E8-mediated phagocytosis after EMF exposure. These results may assist in the development of appropriate microglia-targeted interventions against neurological diseases.

## Methods

### Cell culture and treatments

N9 cells were cultured as described previously [26,27]. Briefly, cells were grown in Iscove’s modified Dulbecco’s medium (IMDM) (HyClone, Logan, UT, USA) with supplementary heat-inactivated fetal bovine serum (5%) (HyClone), 2 mM glutamine, 100 U/ml penicillin, 100 μg/ml streptomycin, and 50 μM 2-mercaptoethanol (Sigma-Aldrich, St Louis, MO, USA). Cells were seeded in 25 cm^2^ flasks (1 × 10^6^ cells/ml), six-well plates (5 × 10^5^ cells/well) or 24-well plates (1.5× 10^5^ cells/well) at 37°C in an incubator (37°C, 5% CO_2_). After 24 h incubation, the medium was replaced with serum-free IMDM supplemented with the following agents for different experiments: a solvent control (tissue culture grade dimethylsulfoxide, Sigma-Aldrich), curcumin (5, 10 and 20 μM, Sigma-Aldrich), recombinant murine MFG-E8 (rmMFG-E8) (100, 500 and 1000 ng/ml), anti-TLR4 (10 μg/ml) (MTS510, HyCult Biotechnology, Uden, Netherlands) and (+)-naloxone (1, 5 and 10 μM) (Sigma-Aldrich). After 1 h incubation, the cells were exposed to EMF for 20 min before they were subjected to staining or to extract proteins. To investigate inflammation in the inhibition of phagocytosis and to ensure the proper concentration for the blocking antibody was used in our experiments, the cells were treated with *Escherichia coli* LPS (200 ng/ml) (Sigma-Aldrich) as a positive control.

### Exposure system

Household appliances, medical equipment and communication systems make extensive use of 2.45 GHz pulsed microwaves [11]. Cells were exposed to a pulsed EMF in an anechoic chamber. The ambient air temperature inside the anechoic chamber was 25°C to 26°C. Pulsed EMF pulses (90 mW) were delivered through a rectangular horn antenna connected horizontally to a handset (Philips PM 7320X, Sivers IMA, Kista, Sweden). The pulse duration was 2 μs and the pulse repetition rate was 500 pulses per second. The exposure was for 20 min to 2.45 GHz pulsed microwaves at an average specific absorption rate of 6 W/kg. During the exposure, the handset was oriented vertically towards the cells at a distance of 90 cm. Flasks and plates with cells were placed in the upper chamber of a Perspex™ water bath (24.5 cm × 21 cm) to maintain a temperature of 37°C during the EMF exposure. The temperature of the medium in the flasks in the upper chamber was maintained at 37°C by circulating heated water through a lower closed chamber. During the sham exposures, control cells experienced the same conditions but without EMF treatment.

### Flow cytometry

Fluorescence-activated cell sorting (FACS) analysis quickly evaluated microglial activation by detecting CD11b expression. Briefly, N9 cells were washed three times in a flow buffer (PBS containing 0.1% (w/v) sodium azide and 1% (w/v) BSA). The cells were then incubated with goat serum (Zhongshan Goldenbridge Biotechnology (ZsBio), Beijing, China) for 20 min at 4°C to block the non-specific binding to Fc receptors. Cells were then spun (5,000 rpm, 5 min) and washed three times in the flow buffer. Subsequently, the cells were incubated with the rat anti-mouse monoclonal antibody to CD11b (1:100) (AbD Serotec, Oxford, UK) or the rat IgG2b isotype control (1:100) (AbD Serotec) for 1 h at 4°C. Goat anti-rat IgG-DyLight®549 (1:200) (AbD Serotec) was used as a secondary antibody. Cells were resuspended in ice-cold flow buffer (250 μl). Quantitative analysis was performed using a FACSCalibur system (BD Biosciences, San Jose, CA, USA).

### Immunofluorescent staining

Our phagocytic model used N9 cells to engulf fluorescent bioparticles (see detailed description below). Round glass cover slips were placed in the well of 24-well plates before seeding N9 cells. After the 20 min EMF exposure, bioparticles (5 × 10^6^ per well) were added to all wells and incubated for different times. Bioparticles were added 1 h before the indicated time points that the cells were tested in the phagocytosis assay. Then the glass cover slips were carefully taken out of the wells at different times and rinsed twice in PBS. After being fixed and permeabilized, the cells were blocked with goat serum (ZsBio) for 20 min at room temperature and washed three times in PBS. The cells were incubated with the rat anti-mouse monoclonal antibody CD11b (1:100) (AbD Serotec) at 37°C for 1 h. After washing three times in PBS, the cells were incubated with the goat anti-rat Alexa Fluor® 488 secondary antibody (1:200) (Molecular Probes, OR, USA) for 1 h at 37°C in the dark. The cover slips with cells were washed three times in PBS and mounted with an aqueous-based anti-fade mounting medium on glass slides. Images of stained cells were captured using a Leica TCS-SP5 confocal laser scanning microscope (Leica, Mannheim, Germany). The confocal images were acquired using Imaris software (version 7.6; Bitplane, Zurich, Switzerland).

### Phagocytosis assay

We performed serial phagocytosis assays on 24-well plates to evaluate the effects of EMF exposure on the phagocytic actions of N9 cells. The appearance of PS on the cellular plasma membrane is used by various phagocytes to recognize apoptotic cells. Therefore, we used a PS-binding Alexa Fluor® 647-conjugated Annexin V bioparticles system to mimic phagocytosis. Briefly, a solution of chloroform and methanol (19:1) was used to make PS solution (10 mg/ml). A three fold amount (v/v) of Alexa Fluor® 647-conjugated Annexin V bioparticles was used to dilute the PS solution; then another 300-fold amount of IMDM (v/v) was added to the mixed PS solution and incubated at 30°C for 30 min. The solution was then sonicated three times for 10 s each at 10 kHz and centrifuged at 500 rpm for 10 min. The bioparticles were added 1 h before the indicated time points that the cells were tested in the phagocytosis assay. Then, a washing step with cold serum-free medium was performed to interrupt any interaction between the phagocytosing microglia and the uningested bioparticles.

The phagocytic ability of N9 cells was evaluated using the fluorescence intensity of the engulfed bioparticles on a plate reader and fluorescence microscopy. The fluorescence plate reader assay was conducted as explained in a previous study [29]. Plates were read on a Tecan Infinite M200 plate reader at an excitation wavelength of 643 nm and an emission wavelength of 665 nm. The fluorescence intensity of the bioparticles in the microscopy assay was calculated as described in previous studies [30-32]. Five replicates were used for each experimental condition. As a negative control, wells with and without cells were used to calculate the background fluorescence, which was then subtracted from the experimental wells. The autofluorescence properties of the negative controls were indistinguishable. The positive control was N9 cells treated with bioparticles alone.

### Enzyme-linked immunosorbent assay of TNF-α, IL-6 and IL-1β

The secretion of TNF-α, IL-6 and IL-1β into the culture supernatant was measured using mouse ELISA kits (eBioscience, San Diego, CA, USA). Briefly, 96-well ELISA plates (NUNC MaxiSorp, eBioscience) were covered with the capture antibody in the coating buffer (1:250, 100 μl/well) overnight at 4°C. The wells were washed five times with wash buffer (PBST: Phosphate-Buffered Saline with 0.05% of Tween-20) and blocked with assay diluent at room temperature for 1 h. Culture media for the different conditions were added to the wells (100 μl/well) and held at 4°C to maximize sensitivity. The next day, the medium was removed and the wells were washed three times in PBST. The detection antibody (1:250, 100 μl/well) was added to the wells. After 1 h incubation and following three washes, the plates were incubated with avidin-HRP (1:250, 100 μl/well) for 30 min at room temperature. Each plate was subsequently incubated with tetramethylbenzidine substrate solution for 15 min. This reaction was stopped with 50 μl of 2 N H_2_SO_4_ stop solution. Absorbance values were measured at 450 nm using a microplate spectrophotometer.

### Nitric oxide determination in culture medium

The production of NO metabolites (nitrates and nitrites) in the culture medium was quantified using a NO detection kit (Nanjing Jiancheng Bioengineering Institute, Nanjing, China). This method uses nitrate reductase to reduce NO^3−^ to NO^2−^ specifically, and the content of NO^2−^ was determined colorimetrically. Briefly, 100 μl of medium was added to each well. Then, 50 μl of nicotinamide adenine dinucleotide and nitrate reductase were added to each well. After 30 min, Greiss reagents I and II (both 50 μl) were added and incubated for 10 min at room temperature. The optical density of each well was determined using a microplate reader with an emission wavelength at 540 nm.

### Western blotting

N9 cells were harvested from flasks and washed twice with ice-cold PBS. The cells were lysed in RIPA lysis buffer (Roche, Penzberg, Germany) containing protease and phosphatase inhibitors (Roche) for 30 min on ice. Lysates were centrifuged at 12,000 rpm for 10 min at 4°C. Some cells were lysed with the Proteo JET™ Cytoplasmic and Nuclear Protein Extraction Kit (MBI Fermentas, MD, USA) to extract nuclear proteins, or the Proteo JET™ Membrane Protein Extraction Kit (MBI Fermentas) to extract membrane proteins. The protein concentration was determined using the bicinchoninic acid method (Beyotime Biotech, Beijing, China). SDS-PAGE (10%) was used to separate the proteins (whole proteins including MFG-E8, 50 μg; membrane proteins including α_v_β_3_, 50 μg; cytoplasmic proteins including pTyr705-STAT3 and STAT3, 50 μg; or nuclear proteins including pTyr705-STAT3 and NF-κB, 20 μg). Protein bands were transferred on to nitrocellulose membranes (Millipore, Bedford, MA, USA) at 4°C. The membranes were blocked in PBS with 5% non-fat milk for 1 h and then incubated with primary antibodies recognizing MFG-E8 (1:500) (Santa-Cruz Biotechnology, Santa Cruz, CA, USA), α_v_β_3_ integrin (1:1000) (Millipore), phospho-STAT3 (p-STAT3) (1:1000) (Cell Signaling Technology, Danvers, MA, USA), STAT3 (1:1000) (Cell Signaling Technology) and p65 NF-κB (1:1000) (Cell Signaling Technology) at 4°C overnight. Membranes were washed four times for 5 min each in tris-buffered saline plus 0.1% Tween-20 (TBST). A fluorescently labeled secondary antibody (IRDye® 800CW donkey anti-mouse IgG (H + L) and IRDye® 680RD donkey anti-rabbit IgG (H + L), (1:5000) (LI-COR, Lincoln, NE, USA) was used to bind the primary antibodies for 1 h at room temperature with gentle shaking in a dark area. After washing in TBST (four times, 5 min each), the protein bands were illuminated and quantified using an Odyssey infrared imaging system (LI-COR). Glyceraldehyde 3-phosphate dehydrogenase (GAPDH) (1:1000) (Cell Signaling Technology) and proliferating cell nuclear antigen (PCNA) (1:500) (Santa Cruz) were used as internal controls for cytoplasmic protein and nuclear protein, respectively. Protein bands were semi-quantified by densitometric analysis using ImageJ 1.46.

### Statistical analysis

Statistical analysis was performed using SPSS software. Each experiment was repeated a minimum of three times and the data expressed as means ± SEM. Statistical differences between the groups were assessed by two-way ANOVA followed by Tukey’s test. Statistical significance was established at *P* < 0.05, unless otherwise indicated.

## Results

### Alteration of phagocytic ability of N9 cells after EMF exposure

**Figure 1:**
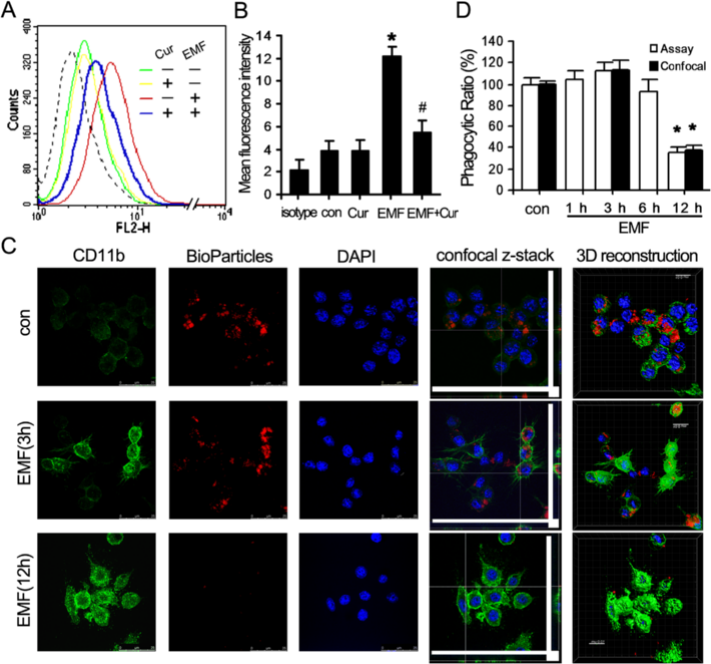
**Reduction of phagocytic ability of N9 cells after EMF exposure.** N9 cells were pretreated with (+) or without (−) curcumin (20 μM) for 1 h and then exposed to 2.45 GHz EMF (+) or sham exposed (−) for 20 min. Untreated cultures were used as controls. **(A)** Representative flow cytometry histograms for CD11b expression at 12 h after EMF exposure. The fluorescence intensity in the FL2-H channel (X-axis) reflects CD11b content. **(B)** Average values of mean fluorescence intensity of CD11b from three independent experiments normalized to the control. **(C)** Confocal and constructed 3D images of N9 cells used as controls, and 3 h and 12 h after EMF exposure. Scale bar: 25 μm. **(D)** Average fluorescence intensity of microspheres ingested per group estimated using the confocal images and multifunctional microplate reader. *P < 0.05 vs the sham-exposed control group; ^#^*P* < 0.05 vs the EMF-exposed group. con, control; Cur, curcumin; DAPI, 4',6-diamidino-2-phenylindole; EMF, electromagnetic field.

EMF exposure dramatically enhanced the expression of CD11b but reduced the phagocytic ability of N9 cells. The expression of CD11b was measured using FACS analysis of the N9 cells cultured in different conditions (Figure 1A). The fluorescent light of the CD11b dye (FL2 channel) generates an electronic signal that can be recorded as high (FL2-H) for the intensity of the staining. The intensity of the staining calculated from FL2-H showed that CD11b expression was quite similar in N9 cells incubated with curcumin and the control group (about 3.8 and 3.9, respectively, Figure 1B). But at 12 h after EMF exposure the expression of CD11b was significantly enhanced (12.2) compared to the control (*P* < 0.05, Figure 1B). Curcumin incubation can effectively neutralize the enhancement of CD11b expression (5.7) after EMF exposure of N9 cells, though the expression level was still higher than for the controls (Figure 1B).

The microscopy assay showed that the normalized phagocytic ratio was 112% at 3 h after EMF exposure, which was similar to that of the control (normalized to 100%) (Figure 1C,D). The normalized ratio dramatically dropped to 37% at 12 h post EMF exposure compared to the control (*P* < 0.05, Figure 1D). Using a fluorescence intensity assay of phagocytosis, we observed that each group of N9 cells ingested a different number of bioparticles. Compared to the phagocytic ratio in control cells, the ratio was slightly higher in cells at 1 h (105%) and 3 h (112%) after EMF treatment (Figure 1D), but it decreased to 92% in cells after 6 h and was significantly reduced after 12 h (36%, *P* < 0.05, Figure 1D). These results indicate that EMF may be a potential risk factor for impaired microglial phagocytosis.

### Reduction of MFG-E8 expression is associated with phagocytic depression

**Figure 2:**
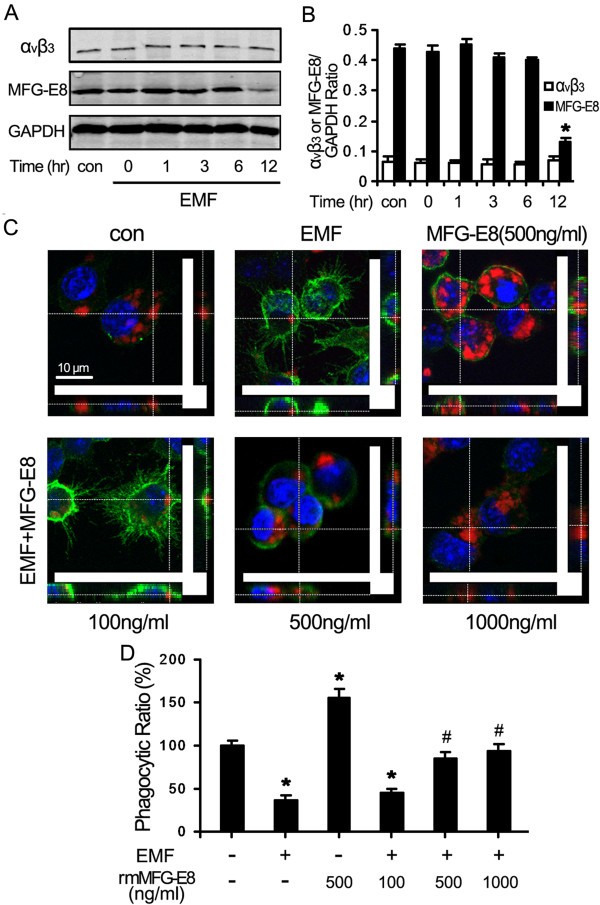
**Improvement in phagocytic ability for EMF-exposed N9 cells with the addition of MFG-E8.** N9 cells were pretreated with (+) or without (−) various concentrations of rmMFG-E8 (100, 500 and 1,000 ng/ml) for 1 h and then exposed to 2.45 GHz EMF (+) or sham exposed (−) for 20 min. **(A)** Levels of MFG-E8 and α_v_β_3_ in total cell lysates and cellular membrane fractions were analyzed using Western blotting at the indicated times for EMF-stimulated N9 cells. Western blot results confirmed downregulation of MFG-E8, but not α_v_β_3_, at 12 h after EMF exposure. **(B)** The MFG-E8 and α_v_β_3_/actin ratios were determined by densitometric analysis. **(C)** rmMFG-E8 pretreatment significantly improved the phagocytic ability of N9 cells at 12 h after EMF exposure. Scale bar: 10 μm. **(D)** Phagocytic ratios were calculated from (C). *P < 0.05 vs the sham-exposed control group; ^#^*P* < 0.05 vs the EMF-exposed group. con, control; EMF, electromagnetic field; MFG-E8, milk-fat globule EGF factor-8; rmMFG-E8, recombinant murine MFG-E8.

EMF exposure significantly depressed the expression of MFG-E8 in N9 cells. We found that the MFG-E8 levels in N9 cells from 0 h to 6 h after EMF treatment were quite similar to those of the controls. The normalized MFG-E8 expression in N9 cells increased about 0.4 fold compared to the internal control (GAPDH) (Figure 2A,B). However, MFG-E8 levels dramatically dropped to about 0.1 fold in N9 cells at 12 h after the EMF exposure (Figure 2A,B). Interestingly, the expression of α_v_β_3_ integrins was not affected by EMF exposure and the normalized fold changes were quite stable (around 0.06 fold increase) (Figure 2A,B). These results indicated that MFG-E8 expression was significantly inhibited at 12 h after EMF exposure.

We tested whether the lower MFG-E8 expression depressed phagocytic ability. The normalized phagocytic ratio dropped to 36% for cells exposed to EMF compared with the sham-exposed controls (normalized to 100%, Figure 2C,D) (*P* < 0.05). When the culture medium was supplied with rmMFG-E8 (500 ng/ml), the ratio increased to 154% (Figure 2C,D), which was significantly higher than for the control (*P* < 0.05). We also found that the phagocytic ability increased with the concentration of rmMFG-E8 (100 ng/ml, 500 ng/ml and 1,000 ng/ml). The phagocytic ratios were 45%, 88% and 94% (Figure 2C,D), respectively.

All of these results demonstrate that EMF exposure can clearly reduce the phagocytic ability of N9 cells but addition of rmMFG-E8 can compensate for the reduction. Our observations confirm that rmMFG-E8 can significantly enhance phagocytosis and restore phagocytic ability in EMF-exposed N9 cells compared to sham-exposed controls. These findings indicate that MFG-E8 plays an important role in phagocytosis.

### Robust pro-inflammatory responses are linked to phagocytic depression

**Figure 3:**
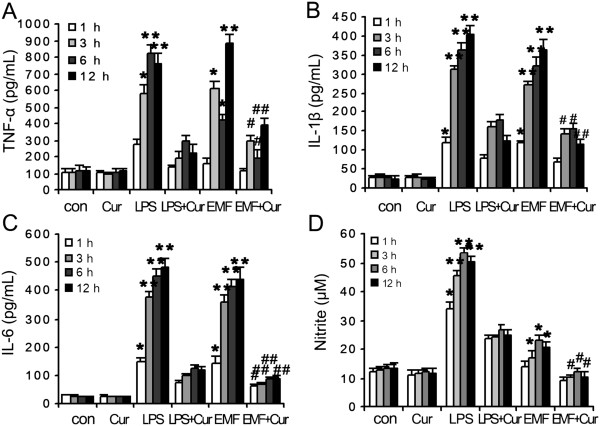
**Inhibition by curcumin of TNF-α, IL-1β, IL-6, and NO in EMF-exposed N9 cells.** N9 cells were pretreated with (+) or without (−) 20 μM curcumin for 1 h, and then stimulated with 200 ng/ml LPS or a 20-min EMF exposure for the indicated times. Levels for each sample were normalized compared to total protein in cell lysates. The concentrations of TNF-α **(A)**, IL-1β **(B)** and IL-6 **(C)** in the medium were determined by ELISA after the cells were treated at the indicated times. **(D)** The amount of nitrite was determined by the Griess reaction. EMF exposure and LPS stimulation significantly increased the secretion of TNF-α, IL-1β and IL-6 and the production of NO in a time-dependent manner from 1 h to 12 h for N9 cells exposed to EMF. Curcumin neutralized the effect in EMF-exposed N9 cells. *P < 0.05, **P < 0.01 vs the sham-exposed control group; ^#^*P* < 0.05, ^##^*P* < 0.01 vs the EMF-exposed group. con, control; Cur, curcumin; EMF, electromagnetic field; IL, interleukin; LPS, lipopolysaccharide; TNF-α, tumor necrosis factor α.

Given the different changes of CD11b expression and phagocytic ability in N9 cells after EMF exposure, we measured levels of TNF-α, IL-1β, IL-6 and NO in cell culture medium supernatants at various times after EMF exposure. ELISA indicated that the secretion of TNF-α, IL-1β and IL-6 was quite low in the sham-exposed control cells and the cells treated with curcumin at all time points (Figure 3A,B,C). The expression of the three cytokines significantly increased in N9 cells after EMF exposure and reached a maximum at 12 h (Figure 3A,B,C). A similar pattern was observed when cells were treated with LPS as a positive control. Dramatically, the expression of the three cytokines dropped to the baseline level in EMF-exposed or LPS-treated N9 cells cultured with curcumin (Figure 3A,B,C). Similarly, no differences were found for the production of nitrite between control cells and curcumin-treated cells (Figure 3D). The levels of nitrite in the media significantly increased after EMF exposure or LPS treatment compared to the control (Figure 3D). The production of NO reduced when curcumin was used to incubate N9 cells after EMF exposure (Figure 3D). In addition, levels of the microglial activation marker CD11b were significantly attenuated by curcumin preconditioning (Figure 1A,B).

**Figure 4:**
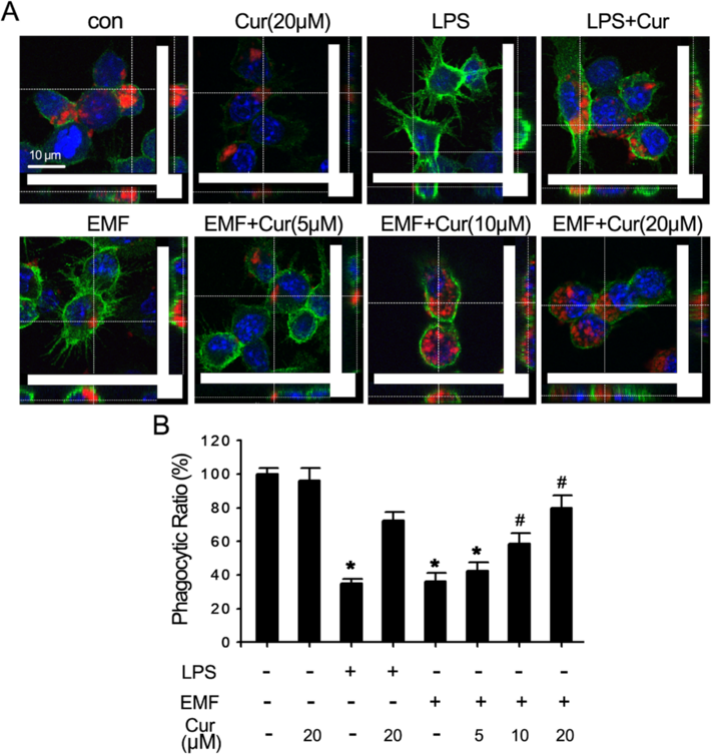
**Amelioration by curcumin of phagocytic ability for EMF-exposed N9 cells.** N9 cells were pretreated with (+) or without (−) various concentrations of curcumin (5, 10 and 20 μM) for 1 h and then exposed to 2.45 GHz EMF (+) or sham exposed (−) for 20 min. **(A)** Curcumin pretreatment concomitantly improves microglial phagocytosis in a dose-dependent manner at 12 h after EMF exposure. No increase in microglial phagocytosis was detected upon curcumin preconditioning in the sham exposure group. Scale bar: 10 μm. **(B)** Quantification of the phagocytic ability of N9 cells was calculated from **(A)**. The phagocytic ratio was significantly improved in the EMF-exposed or 200 ng/ml LPS-treated N9 cells incubated with 10 μM and 20 μM of curcumin. *P < 0.05 vs the sham-exposed control group; ^#^*P* < 0.05 vs the EMF-exposed group. con, control; Cur, curcumin; EMF, electromagnetic field; LPS, lipopolysaccharide.

The phagocytic ratio was quite similar in the controls and N9 cells incubated in curcumin but without EMF exposure (100% vs 96%). The normalized phagocytic ratio at 12 h was as low as 37% for N9 cells treated with EMF and 35% for cells treated with LPS, which was significantly lower than the sham-exposed controls and curcumin-incubated cells (*P* < 0.05, Figure 4A,B). Clearly, at 12 h the phagocytic ratio had improved from 6% to 43% in the EMF-exposed N9 cells supplied with curcumin compared to EMF-treated cells (Figure 4A,B). There was a dose-dependent change in phagocytic ability for N9 cells incubated with curcumin after EMF exposure (Figure 4A,B). These results suggest that the inhibition of microglial pro-inflammatory activation improves the phagocytic ability of N9 cells.

### TLR4 is not involved in chilling the pro-inflammatory response and the amelioration of phagocytosis by curcumin

**Figure 5:**
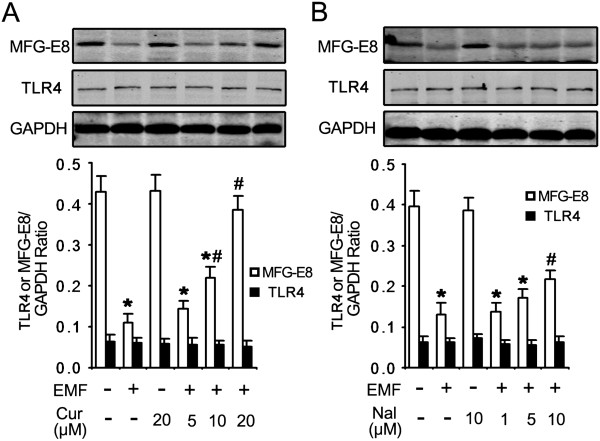
**Restoration of MFG-E8 expression in EMF-exposed N9 cells incubated in curcumin and naloxone.** N9 cells were pretreated with (+) or without (−) various concentrations of curcumin (5, 10 and 20 μM) or naloxone (1, 5 and 10 μM) for 1 h, and then exposed to 2.45 GHz EMF (+) or sham exposed (−) for 20 min. GAPDH antibody was used as a control for equal protein loading. The pro-inflammatory inhibitors curcumin **(A)** and naloxone **(B)** significantly restored the expression of MFG-E8 in a dose-dependent manner at 12 h after EMF exposure but there was no change in TLR4 expression for all groups. Curcumin or naloxone pretreatment alone did not cause significant changes in MFG-E8 expression in the sham exposure group. *P < 0.05 vs the sham-exposed control group; ^#^*P* < 0.05 vs the EMF-exposed group. Cur, curcumin; EMF, electromagnetic field; MFG-E8, milk-fat globule EGF factor-8; Nal, naloxone; TLR4, toll-like receptor 4.

Curcumin alone did not increase the phagocytic ability (Figure 4A,B) or the expression of MFG-E8 (Figure 5A) for the sham exposure group. These results indicate that the improvement of microglial phagocytosis was not directly regulated by curcumin alone. The normalized fold change of protein expression showed that EMF exposure clearly inhibited the expression of MFG-E8 compared to the sham exposure controls (0.11 vs 0.43, Figure 5A,B). An increasing trend of fold change was found for MFG-E8 expression when the EMF-exposed N9 cells were incubated with curcumin (from 0.14 to 0.39, Figure 5A) or naloxone (another anti-inflammatory factor, from 0.14 to 0.22, Figure 5B).

**Figure 6:**
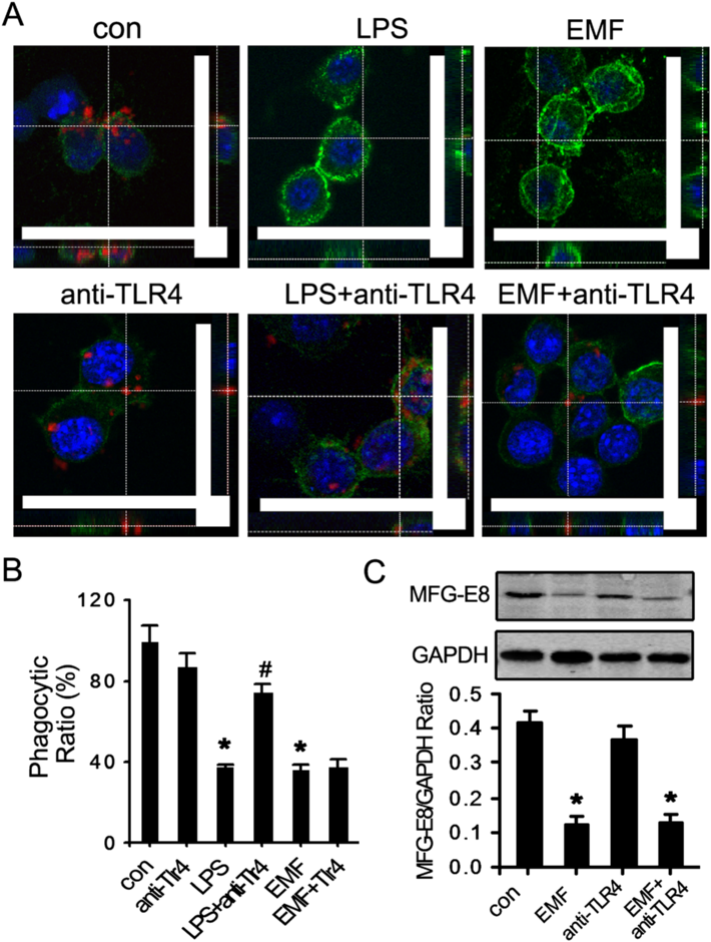
**There was no change in the phagocytic ability of EMF-exposed N9 cells incubated in anti-TLR4 antibody.** Experiments were performed at 12 h after EMF exposure or 200 ng/ml LPS treatment as described above. **(A,B)** Anti-TLR4 antibody (10 μg/ml) pretreatment had no effect on microglial phagocytosis. As a positive control, anti-TLR4 antibody prevented LPS-induced depression of microglial phagocytosis. Scale bar: 10 μm. **(C)** No significant changes in MFG-E8 expression were detected upon anti-TLR4 antibody preconditioning. *P < 0.05 vs the sham-exposed control group; ^#^*P* < 0.05 vs the EMF-exposed group. con, control; EMF, electromagnetic field; LPS, lipopolysaccharide; MFG-E8, milk-fat globule EGF factor-8; TLR4, toll-like receptor 4.

We also tested whether the restoration of MFG-E8-mediated phagocytosis by curcumin was due to the regulation of TLR4. The expression of TLR4 remained at the basal level for each condition, including the curcumin-treated group. The expression of TLR4 was not affected by the addition of curcumin or naloxone, which is a TLR4 inhibitor (Figure 5B). The normalized fold changes were quite similar in all groups (all around 0.50). However, in the TLR4 antibody blocking experiment, the anti-TLR4 antibody did not improve the expression of MFG-E8 (Figure 6C) or ameliorate the phagocytic ratio (Figure 6A,B) in N9 cells treated with EMF. As a positive control, LPS treatment significantly depressed microglial phagocytosis, and this was reversed by the anti-TLR4 antibody (Figure 6A,B). These findings show that the TLR4 pathway might not be involved in the curcumin-mediated improvement of defective phagocytosis.

### The roles of NF-κB and STAT3 in the amelioration of phagocytic ability

**Figure 7:**
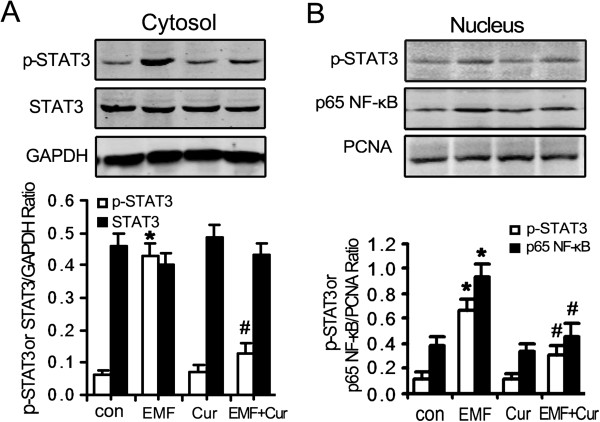
**Involvement of NF-κB and STAT3 signaling in EMF-exposed N9 cells incubated in curcumin.** N9 cells were pretreated with (+) or without (−) curcumin (20 μM) for 1 h, and then exposed to 2.45 GHz EMF (+) or sham exposed (−) for 20 min. Untreated cultures were used as a control. Curcumin pretreatment before EMF exposure significantly inhibits the STAT3 **(A)** and NF-κB **(B)** pro-inflammatory pathways. The nuclear extracts were prepared for Western blot analysis using antibodies against p65 NF-κB or PCNA (used as a loading control). *P < 0.05 vs the sham-exposed control group; ^#^*P* < 0.05 vs the EMF-exposed group. con, control; Cur, curcumin; EMF, electromagnetic field; NF-κB, nuclear factor-κB; PCNA, proliferating cell nuclear antigen; p-STAT3, phospho-STAT3; STAT, signal transducer and activator of transcription.

These findings above show that the improvement of phagocytic ability is not directly regulated by TLR4 through inhibition of the pro-inflammatory responses in N9 cells. Next we studied the anti-inflammatory role of curcumin and revealed that it inhibited the NF-κB and STAT3 pro-inflammatory pathways. Western blot analysis indicated that the addition of curcumin modified the expression of p-STAT3 but not STAT3 in EMF-exposed N9 cells (Figure 7A). The normalized fold change (0.40 to 0.49) of STAT3 was quite stable in the controls and other groups treated with EMF (Figure 7A). But an obvious increase of p-STAT3 expression was found in EMF-treated N9 cells (Figure 7A,B). After the addition of curcumin to the culture medium, the expression of p-STAT3 and NF-κB was chilled down to the baseline level of around 0.30 and 0.46 fold compared to the controls for EMF-exposed N9 cells (Figure 7A,B). These results suggested that curcumin may ameliorate defective microglial phagocytosis via the inhibition of NF-κB activation and STAT3 phosphorylation, but not through alteration of TLR4 signaling.

## Discussion

In the present study, we observed the alteration of the inflammatory response and phagocytosis for EMF-exposed N9 cells. We found significant decreases of phagocytic ability and MFG-E8 expression at 12 h in N9 cells after EMF exposure. The reduction of phagocytosis can be ameliorated in N9 cells incubated with rmMFG-E8. The pro-inflammatory response induced by the EMF exposure was significantly inhibited by curcumin. Interestingly, there was no detectable change in MFG-E8 expression when the TLR4 antibody was supplied to N9 cells, but it was altered by naloxone. Importantly, the STAT3 and NF-κB pro-inflammatory pathways were significantly inhibited by curcumin too. This suggests that curcumin ameliorates EMF-mediated phagocytic depression in N9 cells via the immune regulation of MFG-E8 by the STAT3 and NF-κB pathways.

The primary effects of microglial activation in response to injury and stimuli are the pro-inflammatory and phagocytic responses. It has been reported that pro-inflammatory cytokines act selectively to regulate the different types of microglial phagocytosis [33]. Our results reveal that high levels of pro-inflammatory cytokines were released at 12 h after EMF exposure and this was accompanied by impaired microglial phagocytosis. In the unconjugated bilirubin activated microglial cell model, phagocytosis was similarly downregulated for a certain time period, while considerable amounts of pro-inflammatory cytokines were released [34]. Inflammation-induced aberrant innate immune functions can be prevented by the inhibition of inflammatory processes [17,18,35,36]. As our results demonstrate, inhibition of the EMF-induced pro-inflammatory response using curcumin restored the phagocytic functions of N9 cells. But no increase in phagocytic ability was detected in the control groups after addition of curcumin. Thus, the pro-inflammatory response may be associated with the downregulation of microglial phagocytosis in EMF-treated N9 cells.

EMF exposure can decrease microglial phagocytosis due to downregulated MFG-E8 expression in N9 cells. It has been well known that microglial cells phagocytose apoptotic cells through a process of migration, disfiguration and recognition [37,38]. The recognition of apoptotic cells is mediated by some specific receptors, such as the PS receptor [39] and α_v_β_3_[15]. The soluble PS-binding protein MFG-E8 is required for the recognition and phagocytosis of PS-exposing cells [14,15,40]. MFG-E8 deficiency is accompanied by the induction of pro-inflammatory cytokines and the impaired uptake of apoptotic cells in several inflammatory diseases, including systemic lupus erythematosus [16], sepsis [41] and atherosclerosis [42]. In our study, microglial phagocytosis was decreased in a pro-inflammatory environment at 12 h after EMF exposure, with a parallel decrease in MFG-E8 expression. But the supply of rmMFG-E8 significantly restored microglial phagocytosis. These results suggest that a failure to remove the dying cells dues to a decrease in MFG-E8 expression induced by the pro-inflammatory response. Thus, the pro-inflammatory response appears to cause the non-redundant immune suppression of MFG-E8 expression and microglial phagocytosis after EMF exposure.

Theoretically, TLR-mediated responses can regulate the responses required to eliminate cell debris and promote repair efficiently. Animals that received a single microinjection of TLR2 or TLR4 ligands at the site of sciatic nerve lesions cleared the degenerating myelin faster and recovered earlier than saline-injected control rats [43]. Moreover, the expression of these innate immune receptors by microglia is a natural defense mechanism to prevent β-amyloid peptide accumulation in the CNS [44]. It is recognized that downregulation of MFG-E8 production during sepsis is primarily dependent on activation of the LPS-CD14-TLR4 pathway, and that phagocytosis of apoptotic cells is associated with the TLR4-MFG-E8 pathway [17,45]. However, in our study TLR4 expression was not altered by curcumin or naloxone in EMF-exposed N9 cells. Our results also showed that the anti-TLR4 antibody had no effect on MFG-E8 expression and microglial phagocytosis in EMF-exposed N9 cells. Altogether, the regulation of MFG-E8 expression by the pro-inflammatory response under our experimental conditions was not mediated by TLR4. We propose that MFG-E8-dependent alterations in microglial phagocytosis are not due to the direct regulation of the TLR4 pathway, but rather to the indirect activity of downstream factors related to pro-inflammatory signaling at 12 h after EMF exposure.

Pro-inflammatory cytokines can promote self-propagating cycles of microglial activation by inducing the activation of NF-κB and STAT3, leading to cytokine generation, followed by further activation of NF-κB and STAT3 [46-48]. We reported a possible autocrine loop involving positive feedback between microglial activation and cytokine production [49]. Consistent with the inhibition of pro-inflammatory mediators by curcumin, we observed an anti-inflammatory role of curcumin via inhibition of the STAT3 and NF-κB pro-inflammatory pathways. Therefore, the NF-κB and STAT3 pathways are potentially involved in the anti-inflammatory therapeutic effects of curcumin in a variety of neuropathologies. In support of this, curcumin has been found to block the LPS-mediated induction of cyclooxygenase-2 through inhibition of NF-κB and STAT3 [24,25]. Although the beneficial effects of curcumin can be observed under various experimental conditions, the effects of curcumin in microglial cells exposed to EMF remain to be fully elucidated. Further experiments are required to explore the detailed mechanisms underlying this process. Regardless of the mechanism, the data presented in this study may assist future studies that aim to determine the therapeutic potential of curcumin.

## Conclusions

Our data indicate that pro-inflammatory mediators released by EMF-activated microglia can regulate the expression of MFG-E8, thus decreasing MFG-E8-dependent microglial phagocytosis. The addition of curcumin prevented EMF-induced reductions in microglial phagocytosis and MFG-E8 expression via the inhibition of the pro-inflammatory response. Moreover, curcumin ameliorated phagocytic ability via the prevention of pro-inflammatory activity in an NF-κB- and STAT3-dependent manner without the involvement of TLR4. The modulatory effects of curcumin may have potential for drug discovery for neuroinflammatory disorders.

## Abbreviations

BSA: bovine serum albumin; con: control; CNS: central nervous system; Cur: curcumin; ELISA: enzyme-linked immunosorbent assay; EMF: electromagnetic field; FACS: fluorescence-activated cell sorting; IL: interleukin; IMDM: Iscove’s modified Dulbecco’s medium; LPS: lipopolysaccharide; MFG-E8: milk-fat globule EGF factor-8; NF-κB: nuclear factor-κB; NO: nitric oxide; PBS: phosphate-buffered saline; PCNA: proliferating cell nuclear antigen; PS: phosphatidylserine; p-STAT3: phospho-STAT3; rmMFG-E8: recombinant murine MFG-E8; STAT: signal transducer and activator of transcription; TBST: tris-buffered saline plus 0.1% Tween-20; TLR: toll-like receptor; TNF-α: tumor necrosis factor α.

## Competing interests

The authors declare that they have no competing interests.

## Authors’ contributions

This study is based on an original idea of XSY. GLH, YL and XSY wrote the manuscript. GLH and YL carried out the FACS and confocal double-label immunofluorescence assays. GLH, YL and CHC carried out the Western blotting assay. ML carried out some mediator assays. PG provided the EMF exposure system. ZPY analyzed the data. All authors have read and approved the final manuscript.
